# Determination of intertidal macroalgae community patterns using the power law model

**DOI:** 10.1371/journal.pone.0277281

**Published:** 2022-11-07

**Authors:** Xunmeng Li, Jianqu Chen, Jun Li, Kai Wang, Zhenhua Wang, Shouyu Zhang

**Affiliations:** 1 College of Marine Ecology and Environment, Shanghai Ocean University, Shanghai, China; 2 Key Laboratory of Marine Ecological Monitoring and Restoration Technologies, MNR, Shanghai, China; 3 Engineering Technology Research Center of Marine Ranching, Shanghai Ocean University, Shanghai, China; University of Veterinary and Animal Sciences, PAKISTAN

## Abstract

The spatial heterogeneity of macroalgae in intertidal zones affects the stability of marine ecosystem communities, contributes to the maintenance of coastal biodiversity, and has an essential role in ecosystem and habitat maintenance. We explored the feasibility of applying the power law model to analyze the spatial distribution of macroalgae on Lvhua Island (Zhejiang Province, China) and characterized the intertidal spatial heterogeneity of the macroalgae present. The results showed a strong association between the spatial distribution of macroalgae in the intertidal zone and the power law model (*R*^2^ = 0.98). There was a positive association between species occurrence frequency and the spatial heterogeneity index of macroalgae species. The model also indicated there was macroalgal habitat structure at the site as the spatial heterogeneity within the community was greater than that of random distribution. The power law model reported here provides a new method for macroalgae community ecology research and could be broadly utilized to analyze the spatial pattern of macroalgae in intertidal zones.

## Introduction

Macroalgae are widely distributed and can grow intensively on reef surfaces in marine intertidal zones and form intertidal seaweed beds vital for nearshore marine ecosystems. Intertidal macroalgal communities have crucial ecosystem functions in maintaining biodiversity, water quality, and as sites of primary productivity [1–3].

Spatial heterogeneity describes the variability and spatial distributions of individual species within communities and is an important index for characterizing community ecosystems [4, 5]. Accurately determining the spatial heterogeneity of macroalgae in intertidal zones enhances our understanding of ecosystem spatial distribution characteristics, species diversity, and ecological productivity within macroalgae communities [6]. Studies exploring the spatial heterogeneity of macroalgae communities should start by analyzing small-scale spatial species distribution patterns [7]. The spatial pattern of a small-scale seaweed community is related to the environmental characteristics in the surrounding environment and can better reflect the interspecific and intraspecific relationships [7]. Studying the spatial heterogeneity of macroalgae communities at a small scale also helps to understand the interactions, symbiosis mechanisms, and adaptation strategies within populations [8, 9]. Spatial heterogeneity can also accurately and quantitatively describe species’ horizontal spatial distributions, characterize community composition, and determine dynamic change trends of macroalgae communities [10].

Traditionally, macroalgae community research focuses on species composition, seasonal variations, ecological value, and biodiversity, but fewer report community distributions and landscape patterns in the intertidal zone [11–15]. Taylor (1961) first reported the power law model in the study of plant pathology [16]. More recently, Shiyomi (2001) reported a refined power square model and applied and successfully applied it to the study of the spatial patterns of rice diseases and pests [17, 18].

Among the macroalgae ecological investigation methods, physical sampling and collection is the most commonly used method utilized in macroalgae studies [19]. Physical sampling requires manual placement of quadrats in the survey area during ebb tides and the destructive removal of macroalgae specimens at the holdfast with a shovel. This sampling methodology is challenging, destructive to the local habitat, and undesirable when low abundance and endangered species are present in the sampling site.

In recent years, a new community survey method has been implemented in ecological research, the Binary Method, which reduces the damage caused by physical sampling. The Binary Method comprehensively analyzes community structure and spatial patterns by recording the times of " Presence " (recorded as 1) and " absence " (recorded as 0) of each species in each quadra, and the data are analyzed with the model. This survey method is efficient and straightforward, and has been widely used in grassland and forest system research [20–22].

To provide an enhanced method characterizing the spatial heterogeneity and ecological service values of macroalgae communities, we investigated the structural characteristics of macroalgae communities in an intertidal zone using the power law model.

## Materials and methods

### Study area

Macroalgae community (30°49′30.19″N, 122°37′09.96″E) in the intertidal zone of Lvhua island, (Ma’an islands), Zhejiang Province was assessed for this study, between August 14th–16th, 2020. Macroalgae resources were abundant in the coastal of Lvhua island, that previous studies have shown the greatest abundances and biomass occurring in August, approximately 5.27 kg/m^2^, the dominant species in the intertidal zone include *Sargassum thunbergii*, *Ulva australis*, and *Ishige okamura*e [23].

### Investigation method

**Fig 1:**
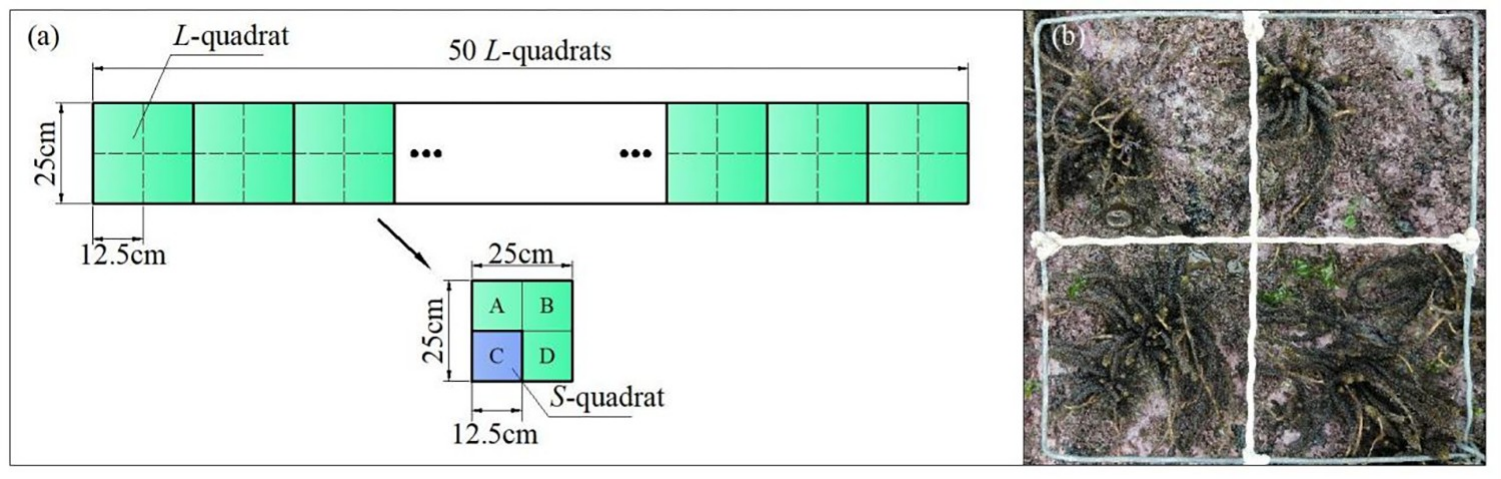
Macroalgae survey methodology and quadrat layout. **(A)** Fifty *L*-quadrats containing 200 *S*-quadrats were successively placed along the transect line, **(B)** the occurrence times of species by the Binary Method.

Three survey transect line was randomly set in the offshore direction of the intertidal zone, from one end, 50 large quadrats (*L*-quadrats) were placed successively along the line. As for the quadrat size, Greig-Smith (1983) and Stewart (1990) believed that the distribution type was related to the spatial scale of the object [24, 25]. The seaweed survey quadrat was 25 × 25 cm [26]. Each *L*-quadrat (25 cm × 25 cm) was divided into four equal 12.5 cm × 12.5 cm quadrats (*S*-quadrats) (**Fig 1A**). Individual species were recorded in the *S*-quadrats, and the occurrence times were recorded in the *L*-quadrats. A presence in the *S*-quadrat was recorded as "1", and absence in the *S-*quadrat was recorded as "0". Species occurrences were counted in the *L*-quadrats, and the presence frequency of each species in all quadrats was calculated [17]. Tillering specimens were counted once, for example, *S*. *thunbergii* (**Fig 1B**).

The frequency of a species (*i*) was

$$p_{i}=\frac{n_{i}}{m}$$ (1)

Where *p*_*i*_ was the frequency of species *i*, *n*_*i*_ was the number of occurrences of species *i* in the *S*-quadrats, *m* was the number of *S*-quadrats in *L*-quadrat, i.e. *m* = 4 in this investigation.

### Power law model for the occurrence frequency of species

*p*_*i*_ was used to denote the frequency of species *i* in *S*-quadrats, and *v*_*i*_ was used to denote the variance in occurrence times of species *i* in *L*-quadrats. The power law model was calculated as: The logarithm of the frequency variance of the random distribution as the abscissa of all species in *L*-quadrats (*x*_*i*_), with the logarithm of the actual frequency variance as the ordinate (*y*_*i*_). A scatter plot was generated showing the corresponding parameter transformations of different macroalgae species, *y*_*i*_ was expressed as the linear regression equation of *x*_*i*_. [17, 27–29]. The formulas were:

$$x_{i}=log\frac{p_{i}(1-p_{i})}{n}$$ (2)

$$y_{i}=log\frac{v_{i}}{n^{2}}$$ (3)

$$y_{i}=\alpha +\beta x_{i}+\varepsilon_{i}(i=1,2,3,4,5\ldots m)$$ (4)

Where, *n* was the number of *S*-quadrats in one *L*-quadrat, and *m* was the total number of macroalgae species identified in along the whole transect, *α* and *β* were the regression coefficients, *ε*_*i*_ was the regression residual of species *i*.

Eq (4) presents the spatial heterogeneity of the whole macroalgae community. *ε*_*i*_ represents the spatial heterogeneity value of species *i* compared with the whole community. Where *ε*_*i*_ is > 0, that species *i* had a greater spatial heterogeneity than the whole community. Where *ε*_*i*_ is < 0, that species *i* had a lower spatial heterogeneity than the whole community.

### Spatial heterogeneity index of different species

In order to express spatial heterogeneity quantitatively of each species, we used *δ*_*i*_ to express a vertical distance line between *y*_*i*_ and *y*_*i*_ = *x*_*i*_, representing the spatial heterogeneity index of species *i*. That is, the distance between the logarithm of the actual frequency variance and the random distribution variance [27]. *δ*_*i*_ was calculated as:

$$\delta_{i}{=y}_{i}-x_{i}=log\frac{v_{i}}{n^{2}}-log\frac{p_{i}(1-p_{i})}{n}$$ (5)

**Fig 2:**
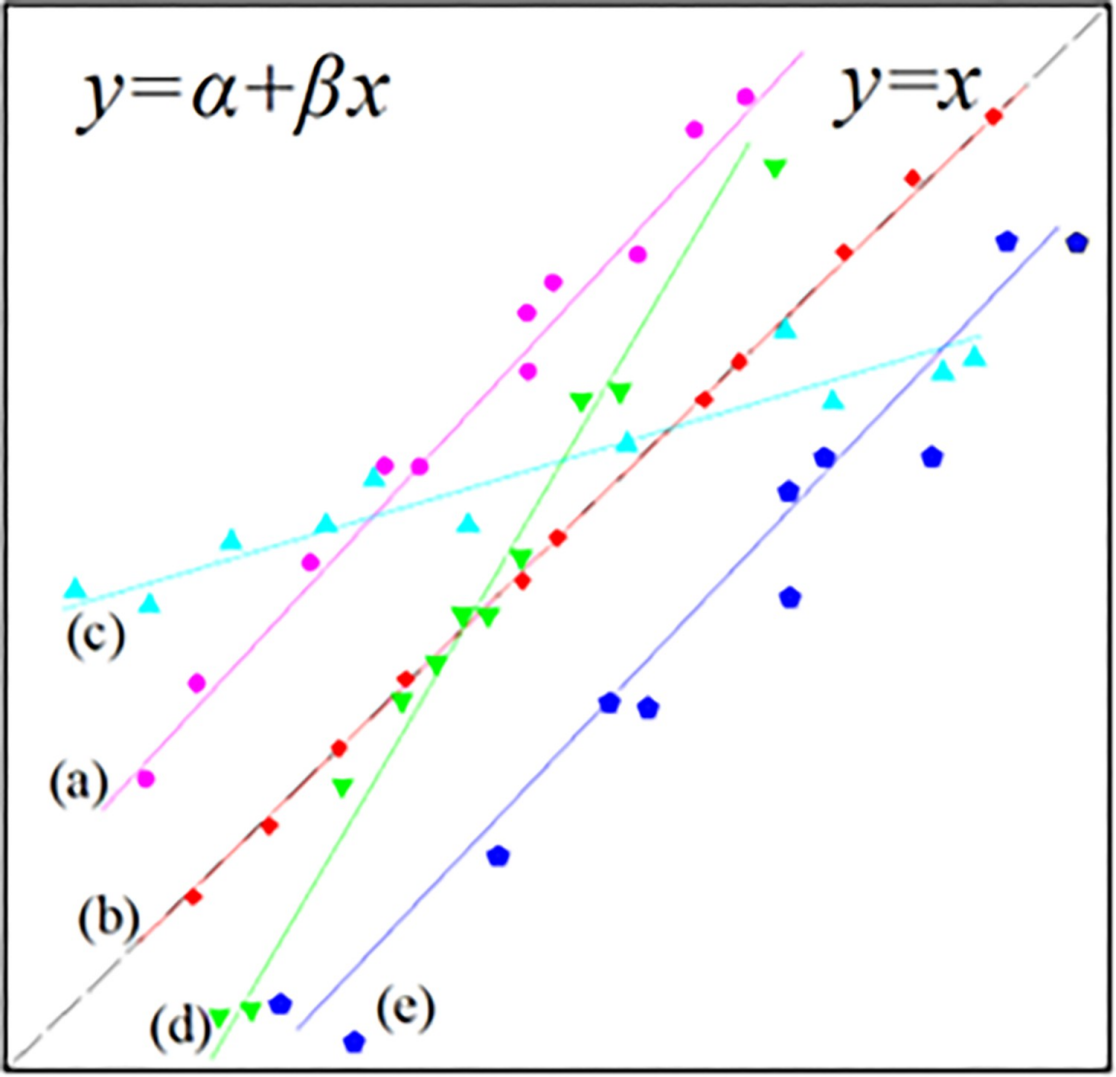
Distribution types generated by the power law model [17]. **(a)** Patchy distribution or cluster distribution, **(b)** random distribution, **(c** and **d)** some species had a patchy distribution, and some species had a uniform distribution, **(e)** uniform distribution. *y = x* line shown as dashed grey line [mostly overplayed by **(b)]**.

The spatial heterogeneity of species *i* can be determined by the vertical distance between the coordinate point (*x*_*i*_, *y*_*i*_) and the line *y* = *x*. The heterogeneity of the whole macroalgae community can be determined by the position of the regression line and the *y* = *x* line (**Fig 2**).

The criteria were as follows:

1. If the heterogeneity index *δ*_*i*_ > 0, the coordinates were above the *y* = *x* line, meaning species *i* had a greater spatial heterogeneity distribution than a random distribution (patchy distribution or cluster distribution).
2. If the heterogeneity index *δ*_*i*_ = 0, the coordinates were on the *y* = *x* line, meaning species *i* had a random distribution.
3. If the heterogeneity index *δ*_*i*_ < 0, the coordinates were below the *y* = *x* line, meaning species *i* had a lower spatial heterogeneity distribution than a random distribution (uniform distribution) [28, 30].

### The spatial heterogeneity index and species diversity index of a community

To express the spatial heterogeneity of the whole community, we considered the spatial heterogeneity of each species (constituting the whole community) and their frequencies. We used the spatial heterogeneity index of the community (*δ*_*c*_) to reflect the heterogeneity and complexity of the spatial pattern of the macroalgae community. The spatial heterogeneity index of each species (*δ*_*i*_) and occurrence frequency (*p*_*i*_) were averaged as described previously [31], and calculated as follows:

$$\delta_{c}=\frac{\sum_{i=1}^{m}p_{i}\delta_{i}}{\sum_{i=1}^{m}p_{i}}$$ (6)

Where *m* was the number of all identified species. The greater the *δ*_*c*_ value, the higher the spatial heterogeneity of the whole community, its discriminant criterion was consistent with *δ*_*i*_.

The diversity index of the macroalgae community was calculated by the Shannon-Wiener diversity index (*H*’), calculated with:

$$H^{\prime }=-\sum_{i=1}^{s}{p\prime }_{i}ln{p\prime }_{i}$$ (7)

Where

$${p\prime }_{i}=\frac{p_{i}}{\sum p_{i}}$$ (8)

### Statistical analysis

The data were analyzed using IBM SPSS Statistics for Windows, version 25 (IBM Corp., Armonk, N.Y., USA). Correlations using the Pearson’s two-tailed test were deemed significant at a 95% confidence level (0.05 cutoff level).

## Results

### The fitness of power law model

**Fig 3:**
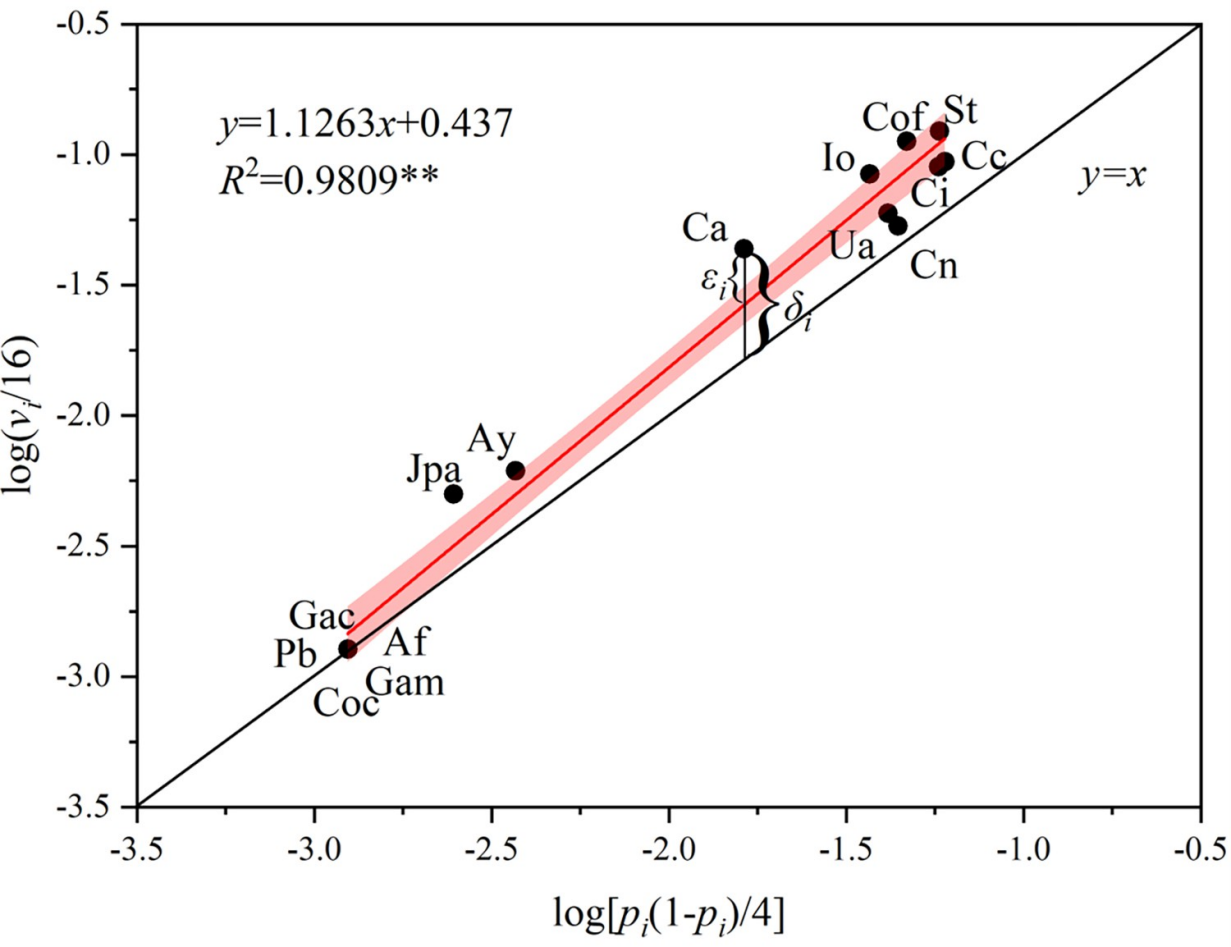
The relationship among macroalgae species occurrence frequencies and the power law model. ** was significantly correlated (p < 0.01). The *y* = *x* line (black line) represents random spatial distribution. Species of macroalgae (black dots) were plotted, and the line of best fit was generated (red line) with the 95% confidence intervals (pink area). The value of spatial heterogeneity of species *i* compared to random distribution in the intertidal zone was calculated as the vertical distance from every point to *y* = *x* straight line (*δ*_*i*_). The value of spatial heterogeneity of species *i* compared to the whole community was calculated as the vertical distance from every point to the fitting line (*ε*_*i*_). Species (black dot) abbreviations: *Callophyllis adnata* (Ca), *Corallina officinalis* (Cof), *Ishige okamurae* (Io), *Sargassum thunbergii* (St), *Jania pedunculata* var. *adhaerens* (Jpa), *Alatocladia yessoensis* (Ay), *Chondria crassicaulis* (Cc), *Chondracanthus intermedius* (Ci), *Ulva australis* (Ua), *Chondrus nipponicus* (Cn), *Grateloupia acuminata* (Gac), *Chondrus ocellatus* (Coc), *Ahnfeltiopsis flabelliformis* (Af), *Gelidium amansii* (Gam), *Petalonia binghamiae* (Pb).

We plotted the relationship between the occurrence frequency of each species and the power law model [32]. The line of best fit was calculated along with the coefficient of determination (*R*^2^ = 0.9809) (**Fig 3**). The Pearson correlation test showed that *x*_*i*_ and *y*_*i*_ were significantly correlated (p < 0.01), indicating that the power law model accurately determined the spatial distribution characteristics for each species in the macroalgae community.

All the data points in Fig 3 were located above the *y* = *x* line, indicating that the distribution of macroalgae in the intertidal zone exhibit a level of spatial heterogeneity greater than would be expected by random distribution alone. The regression line of the power law model was also above *y* = *x* line, indicating that the community displays a heterogeneous distribution as a whole.

### Spatial pattern of whole macroalgae community

Fifteen species of macroalgae were identified in the survey area. Species of the phylum Rhodophyta were most representative (*n* = 11), followed by the phylum Ochrophyta (*n* = 3), and the phylum Chlorophyta with a single species (**Table 1**). The most abundant and high-frequency species included the Chlorophyte *U*. *australis* and the Rhodophytes *Corallina officinalis* and *Chondria crassicaulis*. The spatial heterogeneity index of the macroalgae community (*δ*_*c*_) was 0.245, and the Shannon-Wiener diversity index (*H*’) was 1.964.

**Table 1:** Identified macroalgae species and their spatial patterns.

Species	Abbreviation	*P*_*i*_	*Ɛ*_*i*_	*δ*_*i*_
*Jania pedunculata* var. *adhaerens*	Jpa	0.0100	0.1976	0.3054
*Alatocladia yessoensis*	Ay	0.0150	0.0915	0.2213
*Chondria crassicaulis*	Cc	0.6000	-0.0881	0.1946
*Grateloupia acuminata*	Gac	0.0050	-0.0591	0.0110
*Chondrus ocellatus*	Coc	0.0050	-0.0591	0.0110
*Chondrus nipponicus*	Cn	0.2300	-0.1866	0.0795
*Corallina officinalis*	Cof	0.7500	0.1101	0.3792
*Ahnfeltiopsis flabelliformis*	Af	0.0050	-0.0591	0.0110
*Gelidium amansii*	Gam	0.0050	-0.0591	0.0110
*Callophyllis adnata*	Ca	0.0700	0.2155	0.4267
*Chondracanthus intermedius*	Ci	0.3600	-0.0881	0.1923
*Petalonia binghamiae*	Pb	0.0050	-0.0591	0.0110
*Sargassum thunbergii*	St	0.3650	0.0461	0.3268
*Ishige okamurae*	Io	0.1800	0.1027	0.3587
*Ulva australis*	Ua	0.7900	-0.1050	0.1574

Table 1 shows the spatial heterogeneity indices for the identified species. The species *Grateloupia acuminata*, *Chondrus ocellatus*, *Ahnfeltiopsis flabelliformis*, *Gelidium amansii*, and *Petalonia binghamiae* had the lowest scores of 0.011, indicating these species exhibit random distributions.

### The relationship between occurrence frequency and spatial heterogeneity index

**Fig 4:**
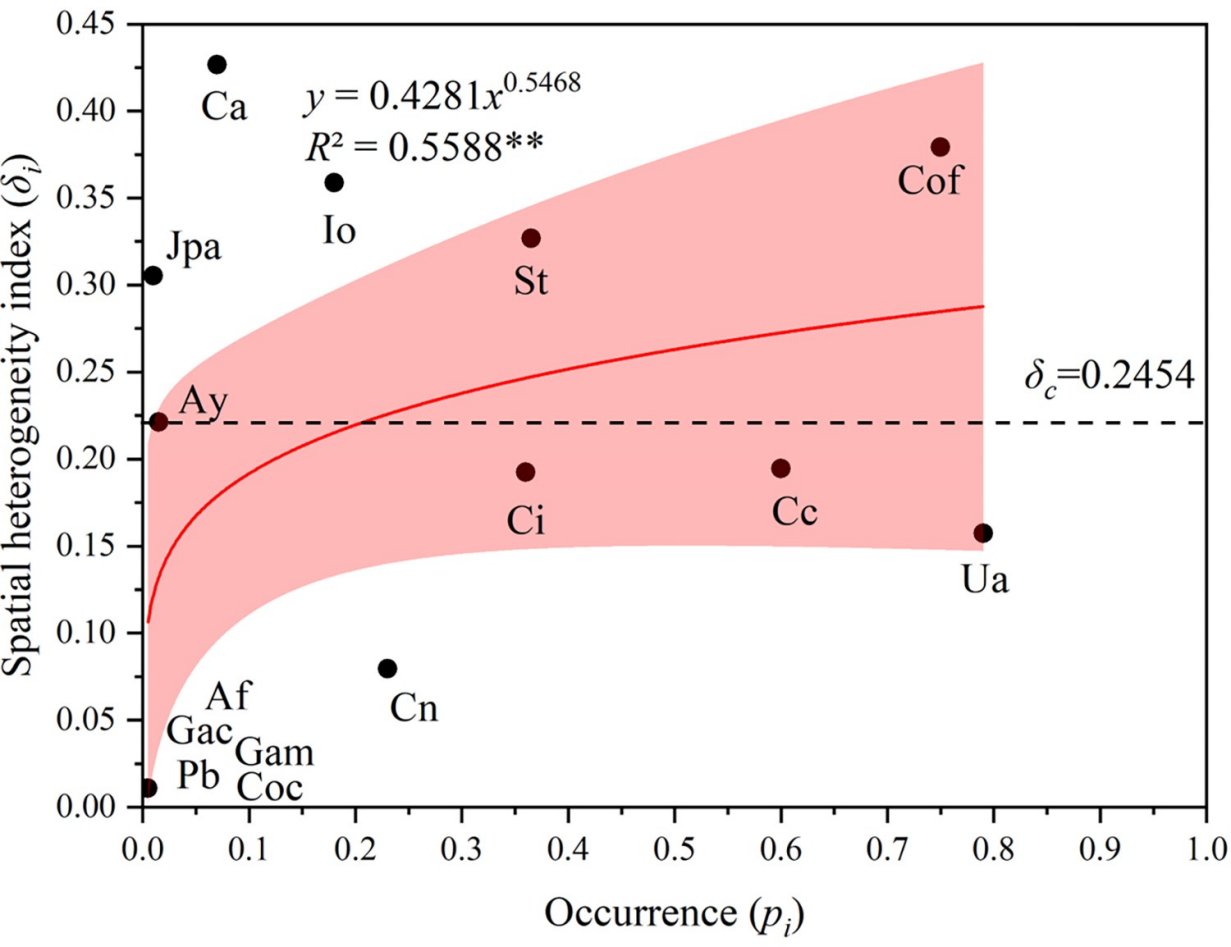
The relationship between occurrence and spatial heterogeneity index. ** was significantly correlated (p < 0.01). The line of best fit (red line) was generated with 95% confidence intervals (pink area). Individual macroalgae species (black dots) were plotted (abbreviations defined below). The overall spatial heterogeneity index of the seaweed community (dotted line) was calculated (*δ*_*c*_ *=* 0.2454). Species (black dot) abbreviations: *Callophyllis adnata* (Ca), *Corallina officinalis* (Cof), *Ishige okamurae* (Io), *Sargassum thunbergia* (St), *Jania pedunculata* var. *adhaerens* (Jpa), *Alatocladia yessoensis* (Ay), *Chondria crassicaulis* (Cc), *Chondracanthus intermedius* (Ci), *Ulva australis* (Ua), *Chondrus nipponicus* (Cn), *Grateloupia acuminata* (Gac), *Chondrus ocellatus*, (Coc) *Ahnfeltiopsis flabelliformis* (Af), *Gelidium amansii* (Gam), *Petalonia binghamiae* (Pb).

A scatter plot was generated taking the occurrence frequency (*p*_*i*_) of all species in the community as the abscissa and the spatial heterogeneity index (*δ*_*i*_) as the ordinate, the line of best fit relationship was fitted (**Fig 4**). The spatial heterogeneity index of each species rose with an increase in occurrence frequency and was positively correlated (*R*^2^ = 0.5588). The spatial heterogeneity index (*δ*_*i*_) for all species was greater than 0, indicating that these intertidal macroalgae have a higher spatial pattern than random distribution. The macroalgae species with the highest spatial heterogeneity indices were *Callophyllis adnata*, *C*. *officinalis*, and *I*. *okamurae* were plotted above the dotted line *δ*_*c*_ *=* 0.2454. It showed that they made the greatest contributions to the overall spatial heterogeneity within the community. *Petalonia binghamiae*, *Gelidium amansii*, and *Ahnfeltiopsis flabelliformis* were located below the straight line, indicating these species made a smaller contribution to the overall spatial heterogeneity within the community.

## Discussion

Traditional research on intertidal macroalgae communities typically begins by considering species composition and diversity, seasonal population variations, and ecological niches [14]. The most common research indices include the Shannon-Wiener index, Margelef’s index and Pielou’s index, but the aspects of spatial patterns were rarely explored [14]. Macroalgae communities exhibit patchy distributions but determining local macroalgae community structures can aid more appropriate analyzes of species interspecific relationships and help to characterize the stability of intertidal macroalgae communities at an ecosystem level.

**Fig 5:**
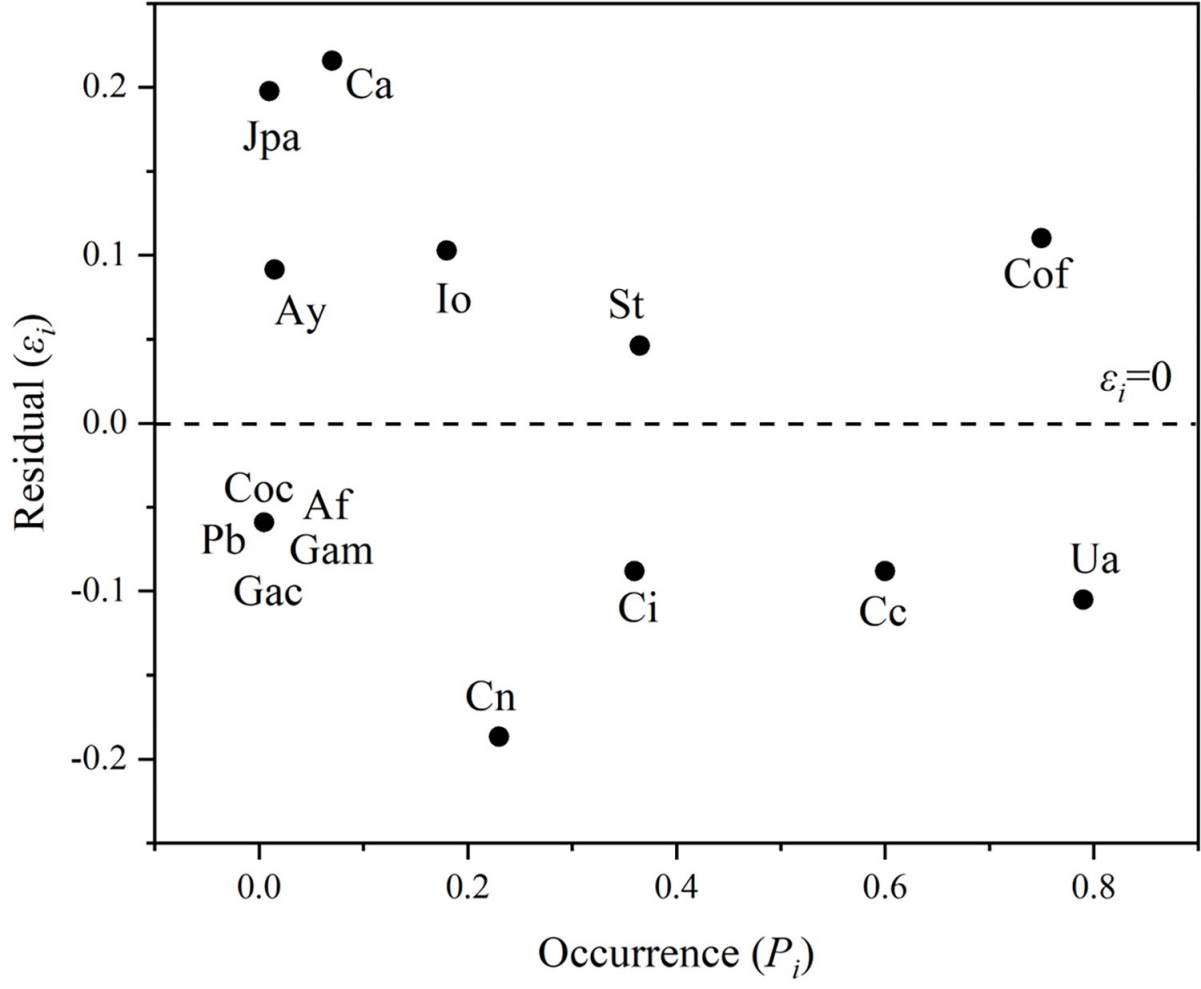
The residuals between the occurrence of individual species and the spatial heterogeneity within the macroalgae community. *Chondrus ocellatus* (Coc), *Chondrus nipponicus* (Cn), and *Chondria crassicaulis* (Cc) were all located below the dashed line (*ε*_*i*_ = 0), indicating that the spatial heterogeneity of each species was less than that of the community, reducing the overall heterogeneity of the community. *Jania pedunculata* var. *adhaerens* (Jpa), *Callophyllis adnata* (Ca), and *Corallina officinalis* (Cof) were located above the straight line, indicating that their spatial heterogeneity was greater than that of the community and that they have a role in increasing the overall heterogeneity of community structure. *C*. *officinalis* (Cof) and *Ulva australis* (Ua) appeared most frequently, which had a great impact on the overall spatial heterogeneity of the community. Species (black dot) abbreviations: *Ishige okamurae* (Io), *Sargassum thunbergia* (St), *Alatocladia yessoensis* (Ay), *Chondracanthus intermedius* (Ci), *Grateloupia acuminata* (Gac), *Ahnfeltiopsis flabelliformis* (Af), *Gelidium amansii* (Gam), *Petalonia binghamiae* (Pb).

In our study, the line of best fit (*R*^2^ = 0.981) (**Fig 3**) and the residuals (**Fig 5**) were mainly below 0.2. These data indicate that the power law model can accurately determine the spatial distribution of the occurrence frequencies of various macroalgae species in the community [18, 33, 34]. The line of best fit in the power law model was above the *y* = *x* line, indicating that this intertidal macroalgae community had a greater spatial heterogeneity than would be explained by random distribution alone.

The growth of macroalgae is greatly affected by environmental factors such as wave action and light, both of which are strongly influenced further by water depth [35]. There is a large gap in the distribution of macroalgae species along the vertical shoreline. We suggest that when studying the spatial patterns of macroalgae on a small scale, studies should consider the distribution characteristics of communities at the same horizontal level.

Macroalgae are distributed on the reef surface, and their growth and development are affected by physical factors, e.g., water temperature, bottom type, and sediment, chemical factors, e.g., nutrients, dissolved oxygen, and pH, and biological factors, e.g., anthropogenic factors, interspecific competition, and herbivores [36]. Among them, temperature is an important factor affecting the nutrient absorption, and has a significant impact on the respiration and the enzyme activity in the dark reaction of photosynthesis of seaweed, and macroalgae growing in the cold zone have good adaptability to low temperature, while living in the tropics can adapt to higher water temperature [37]. PH affects the active absorption by changing the enzyme activity of macroalgae, thus affecting various metabolic processes [36]. Generally, the nutrient absorption rate accelerates with the increase of nutrient concentration in water [38]. Human harvesting activities affect the total amount of spores released in the coming year [39]. Because different seaweed eaters have different tolerance to wave disturbance, macroalgae can be survived in the barren area of sea urchin [40]. This research just analyzed the distribution of macroalgae in the intertidal zone from the perspective of landscape ecology of LvHua island, and does not analyzed the influence mechanism of environmental factors on the distribution specifically. In order to fully reveal the effectiveness of the power law model in analyzing the distribution of seaweed in the intertidal zone, the distribution of macroalgae in the intertidal zone should be deep studied at different sites by different temperature, slope, and wave disturbance conditions.

## Conclusion

We used the power law to study the distribution characteristics of a macroalgae community in an intertidal zone, and have described an alternative method to traditionally destructive macroalgae sampling and collection. Our method is novel, relatively simple to conduct, and could play an important role in protecting macroalgae resources. Following our data analysis, the scatter plots show the distribution patterns of spatial heterogeneity at a community level, as well as each individual species’ contribution to community structure. We have shown that the power law model can effectively characterize the species composition, diversity index and the contribution of each species to spatial heterogeneity within the community. In future studies, by combining the technology of drones, we can quickly evaluate macroalgae resources in intertidal zones without physical collection and the destruction of macroalgae communities. This investigation method can minimize the use of damaging sampling techniques in macroalgae habitats and greatly reduces the workload of investigators. Although a preliminary study, this method has the potential to enhance macroalgal community research considerably. We suggest that the power law model can be used as a new method to study the spatial pattern of macroalgae communities in the intertidal zone.

## Supporting information

S1 Table Distribution characteristics. (XLSX)

S1 Fig Investigation method. (PDF)

S2 Fig Distribution types. (PDF)

S3 Fig Statistical results. (PDF)

S4 Fig Spatial heterogeneity index. (PDF)

S5 Fig Residuals. (PDF)
