# Supplementary figures and images for: Determination of intertidal macroalgae community patterns using the power law model
Source: PLoS One. 2022 Nov 7;17(11):e0277281. doi: 10.1371/journal.pone.0277281 (PMC9639843; doi:10.1371/journal.pone.0277281)

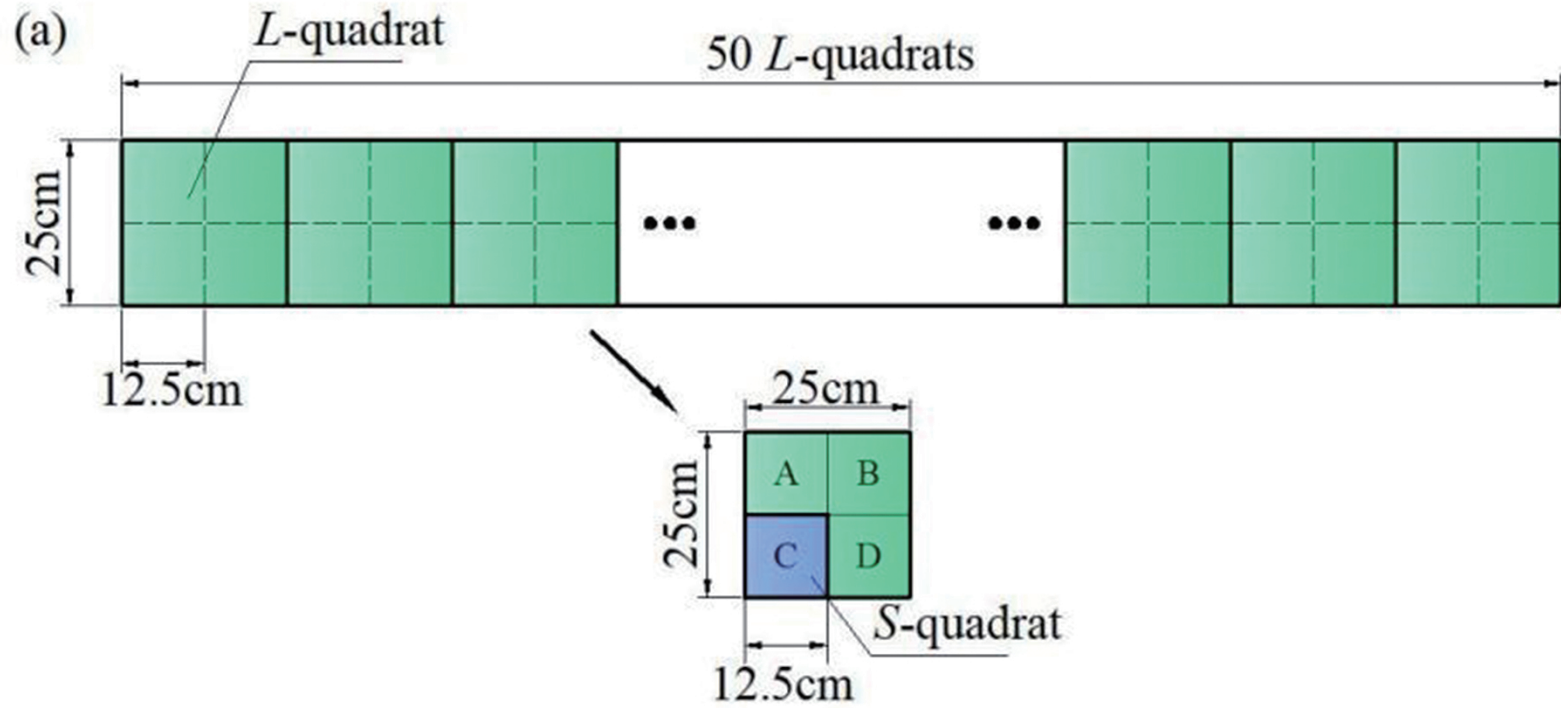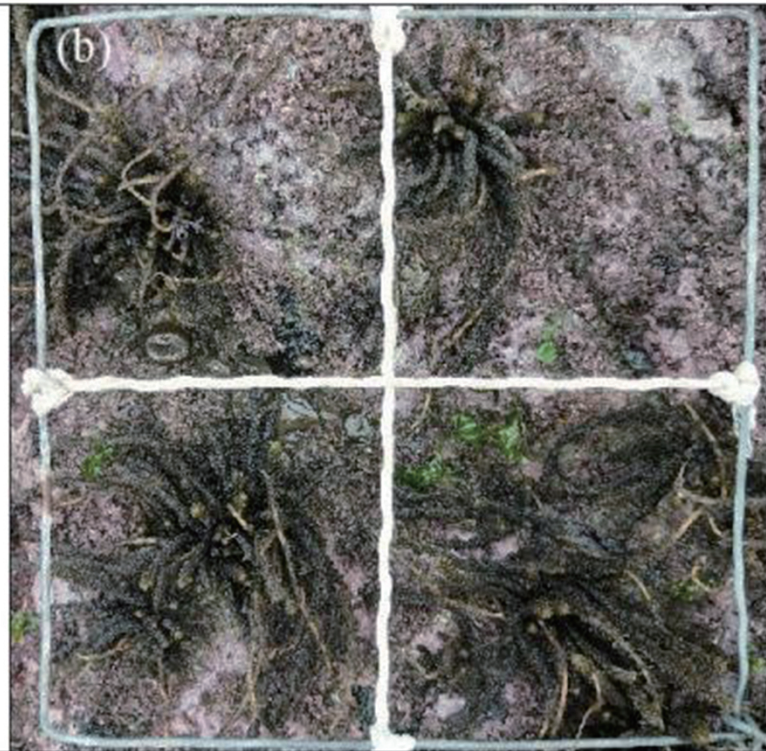

Supplement: S1 Fig — (PDF) [file pone.0277281.s002.pdf]

$$y = \alpha + \beta x$$

$$y = x$$

(c)

(a)

(b)

(d)

(e)

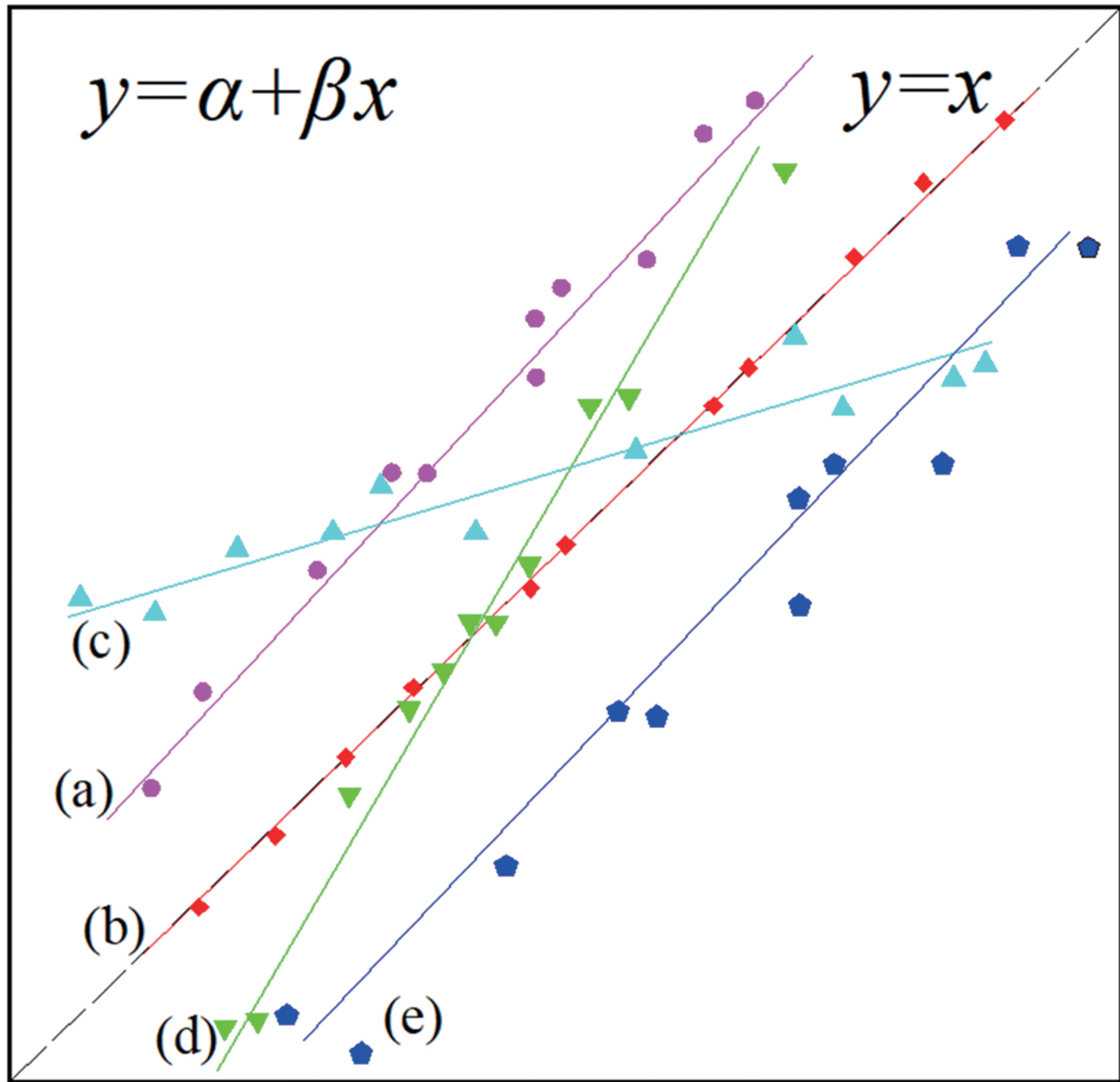

Supplement: S2 Fig — (PDF) [file pone.0277281.s003.pdf]

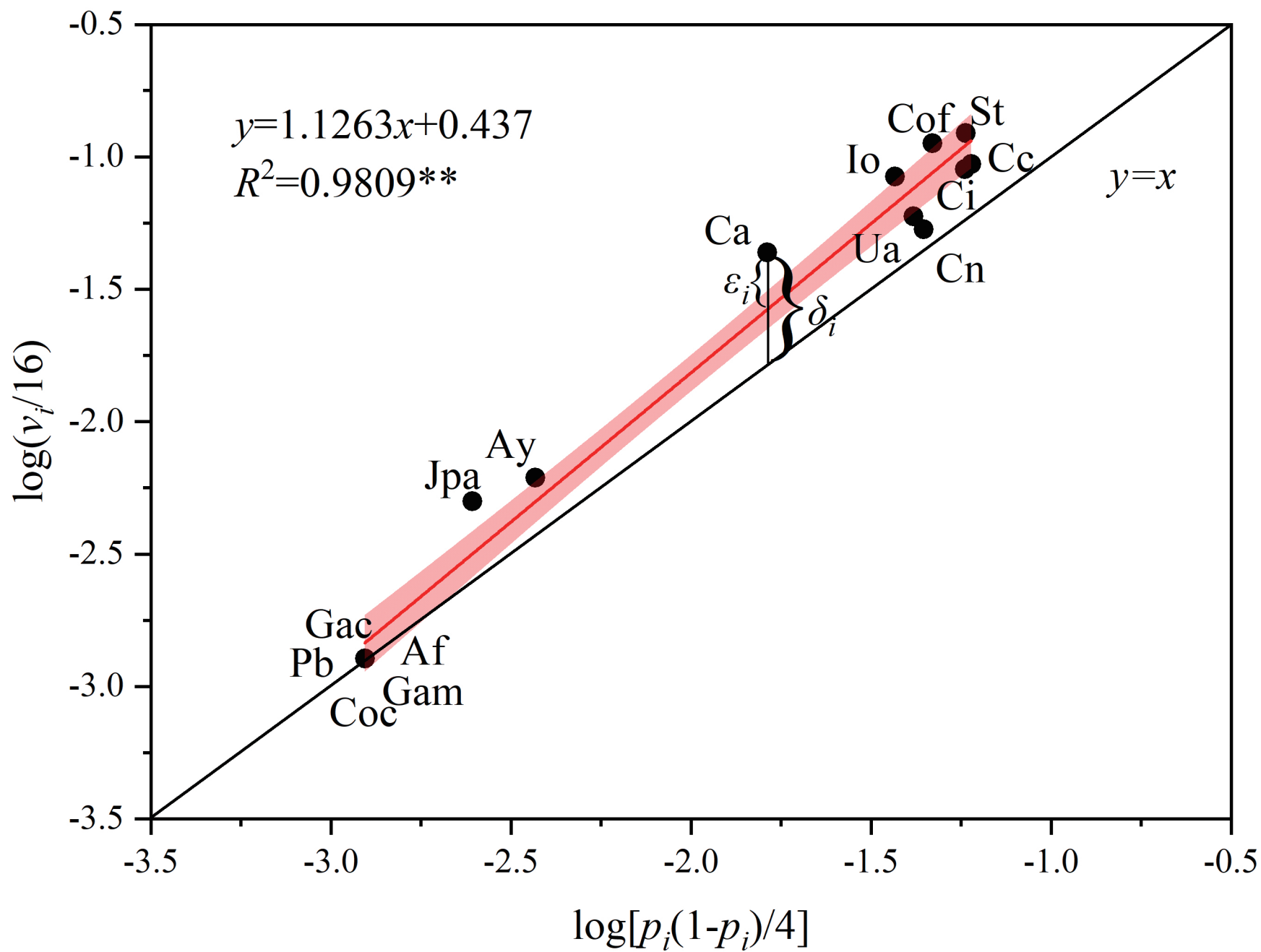

Supplement: S3 Fig — (PDF) [file pone.0277281.s004.pdf]

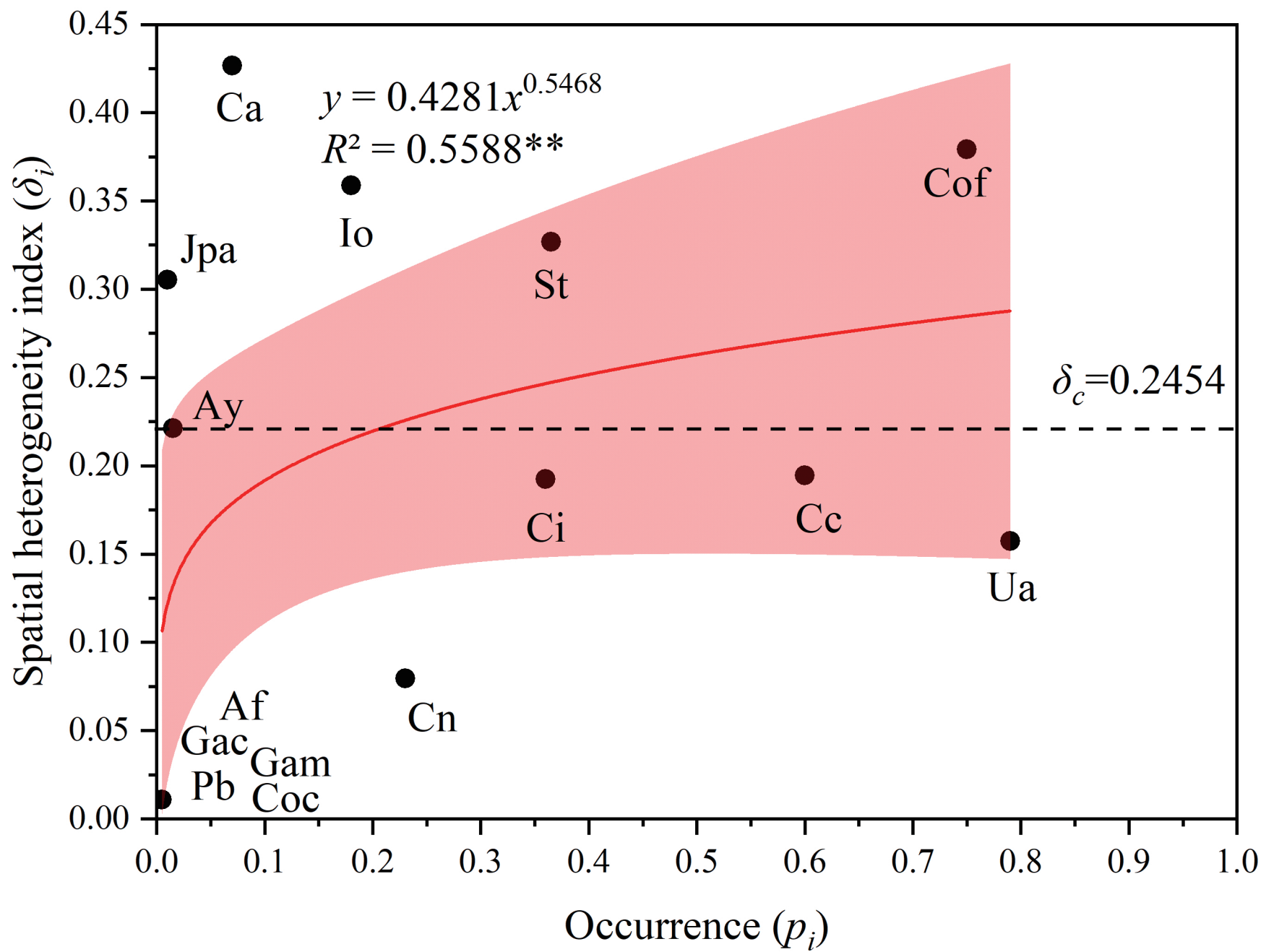

Supplement: S4 Fig — (PDF) [file pone.0277281.s005.pdf]

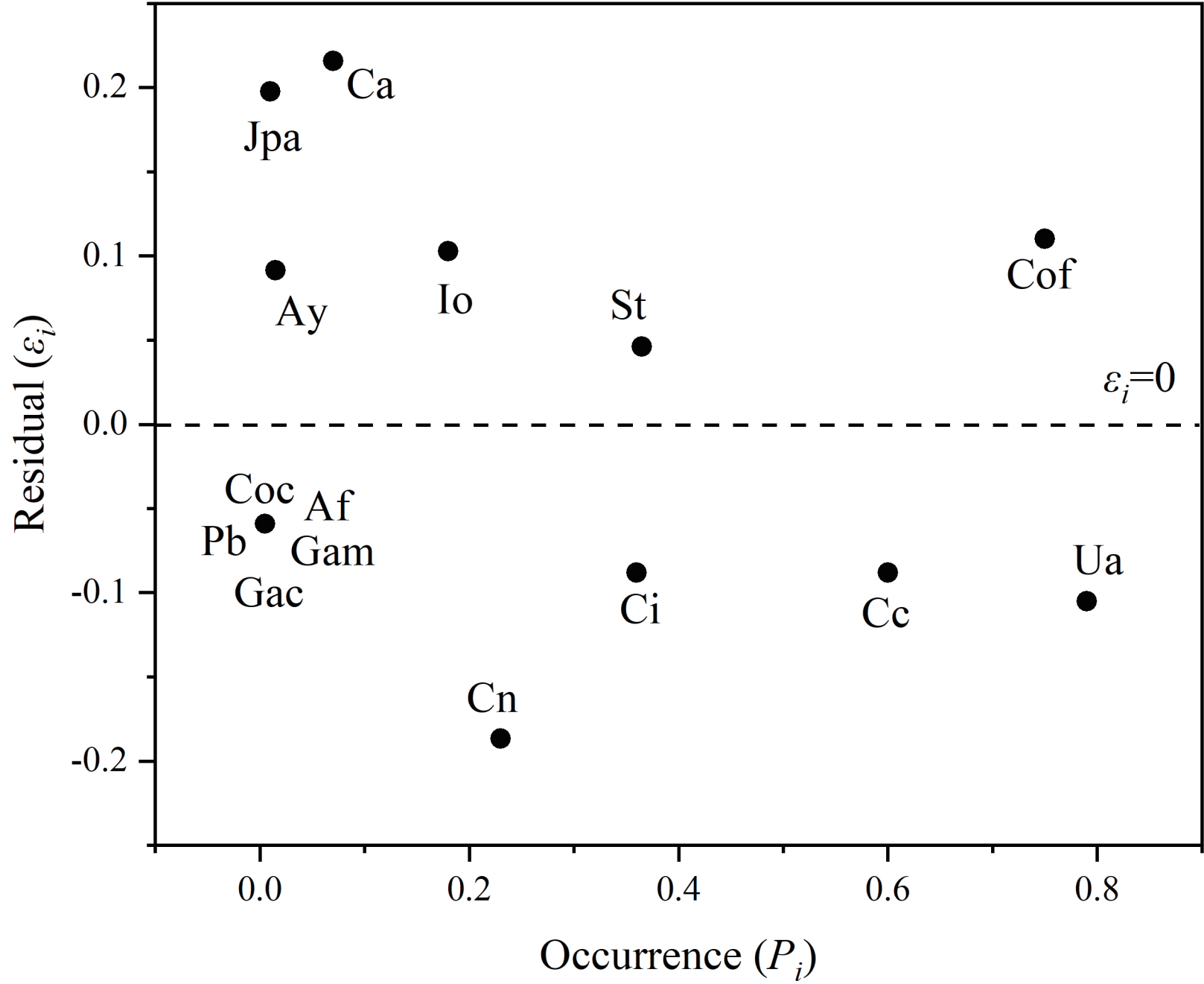

Supplement: S5 Fig — (PDF) [file pone.0277281.s006.pdf]
